# Association Between Early Oral β-Blocker Therapy and In-Hospital Outcomes in Patients With ST-Elevation Myocardial Infarction With Mild-Moderate Heart Failure: Findings From the CCC-ACS Project

**DOI:** 10.3389/fcvm.2022.828614

**Published:** 2022-04-15

**Authors:** Miao Wang, Jing Liu, Jun Liu, Yongchen Hao, Na Yang, Tong Liu, Sidney C. Smith, Yong Huo, Gregg C. Fonarow, Junbo Ge, Louise Morgan, Changsheng Ma, Yaling Han, Dong Zhao, Siyan Zhan

**Affiliations:** ^1^Department of Epidemiology and Biostatistics, School of Public Health, Peking University, Beijing, China; ^2^Department of Epidemiology, Beijing Anzhen Hospital, Beijing Institute of Heart, Lung and Blood Vessel Diseases, Capital Medical University, Beijing, China; ^3^Department of Cardiology, Beijing Anzhen Hospital, Capital Medical University, Beijing, China; ^4^Division of Cardiology, University of North Carolina at Chapel Hill, Chapel Hill, NC, United States; ^5^Department of Cardiology, Peking University First Hospital, Beijing, China; ^6^Division of Cardiology, Geffen School of Medicine at University of California, Los Angeles, Los Angeles, CA, United States; ^7^Department of Cardiology, Zhongshan Hospital, Shanghai Institute of Cardiovascular Diseases, Fudan University, Shanghai, China; ^8^International Quality Improvement Department, American Heart Association, Dallas, TX, United States; ^9^Cardiovascular Research Institute and Department of Cardiology, General Hospital of Northern Theater Command, Shenyang, China

**Keywords:** β-blocker, early treatment, heart failure, myocardial infarction, in-hospital outcomes

## Abstract

**Background:**

There are limited data available on the impact of early (within 24 h of admission) β-blocker therapy on in-hospital outcomes of patients with ST-elevation myocardial infarction (STEMI) and mild-moderate acute heart failure. This study aimed to explore the association between early oral β-blocker therapy and in-hospital outcomes.

**Methods:**

Inpatients with STEMI and Killip class II or III heart failure from the Improving Care for Cardiovascular Disease in China project (*n* = 10,239) were enrolled. The primary outcome was a combined endpoint composed of in-hospital all-cause mortality, successful cardiopulmonary resuscitation after cardiac arrest, and cardiogenic shock. Inverse-probability-of-treatment weighting, multivariate Cox regression, and propensity score matching were performed.

**Results:**

Early oral β-blocker therapy was administered to 56.5% of patients. The incidence of the combined endpoint events was significantly lower in patients with early therapy than in those without (2.7 vs. 5.1%, *P* < 0.001). Inverse-probability-of-treatment weighting analysis demonstrated that early β-blocker therapy was associated with a low risk of combined endpoint events (HR = 0.641, 95% CI: 0.486–0.844, *P* = 0.002). Similar results were shown in multivariate Cox regression (HR = 0.665, 95% CI: 0.496–0.894, *P* = 0.007) and propensity score matching (HR = 0.633, 95% CI: 0.453–0.884, *P* = 0.007) analyses. A dose-response trend between the first-day β-blocker dosages and adverse outcomes was observed in a subset of participants with available data. No factor could modify the association of early treatment and the primary outcomes among the subgroups analyses.

**Conclusion:**

Based on nationwide Chinese data, early oral β-blocker therapy is independently associated with a lower risk of poor in-hospital outcome in patients with STEMI and Killip class II or III heart failure.

## Introduction

Cardiovascular diseases cause a serious health burden (1), and the most harmful cardiovascular disease is ST-elevation myocardial infarction (STEMI), which has an acute onset and high mortality rate. Patients with both STEMI and heart failure (HF) have a three- to four-fold greater risk of in-hospital death than those with STEMI without HF (2).

To enable the timely treatment of patients with STEMI to reduce mortality and improve the prognosis, the STEMI treatment and diagnosis guidelines provide a series of treatment recommendations for STEMI and constant updates based on new evidence. β-blocker therapy reduces oxygen consumption, prolongs the diastolic period, and increases blood supply to the heart by reducing the myocardial contractility, heart rate, and blood pressure, and further decreases the myocardial infarction size and mortality risk. Thus, the guidelines recommend the initiation of oral β-blocker therapy within 24 h of the acute onset of STEMI if there are no contraindications, but recommend delayed or reduced-dose oral β-blocker therapy for patients with STEMI with HF (3, 4).

The recommendation of early β-blocker therapy (within 24 h of admission) in patients with STEMI without HF is based on the results of randomized controlled trials that excluded patients with STEMI with significant HF (5, 6). However, there is lack direct evidence of the effectiveness of delaying or reducing the dosage of β-blocker therapy for patients with STEMI with acute HF. A *post hoc* analysis of 45,852 patients with acute myocardial infarction (AMI) with Killip class I–III showed that early β-blocker therapy did not reduce the incidence of combined endpoint events (death, re-infarction, and cardiac arrest) in the acute phase (28 days after onset) compared with no therapy, but early therapy did reduce the incidence of combined endpoint events in the group of patients excluding those with a length of stay of ≤1 day (7). However, the study did not analyze the effect of early β-blocker therapy in patients with mild-moderate HF (Killip class II or III) after excluding patients with a length of stay of ≤1 day.

A study of the in-hospital outcomes of patients with non-ST-elevation myocardial infarction (NSTEMI) showed that early β-blocker therapy was initiated in 62% of those with Killip class II and III HF, and the risk of in-hospital death was lower in patients with NSTEMI who received early β-blocker therapy than in those without early therapy (OR = 0.39, 95% CI: 0.23–0.68) (8). The 2021 ESC Guidelines for the diagnosis and treatment of acute and chronic heart failure recommend cautious initiation of in-hospital β-blocker therapy for patients with acute HF, but do not provide levels of evidence and recommendation or cite relevant research (9).

Overall, there is insufficient evidence regarding the effect of early β-blocker therapy on the outcomes of patients with AMI with mild-moderate HF (Killip class II or III); in particular, there is a lack of studies focusing on patients with STEMI with mild-moderate HF. Based on the data of the Improving Care for Cardiovascular Disease in China (CCC) project, the present study aimed to evaluate the utilization rate of early in-hospital oral β-blocker therapy in patients with STEMI with mild-moderate HF, and evaluate the association of such early therapy with in-hospital outcomes (combined endpoint composed of all-cause death, resuscitated cardiac arrest, and cardiogenic shock).

## Materials and Methods

### Participants

The study participants were from the CCC project, which is a nationally registry study jointly conducted by the Chinese Society of Cardiology and the American Heart Association. The CCC project was started in December 2014 and included patients with a discharge diagnosis of acute coronary syndrome (ACS) (including STEMI, NSTEMI, and unstable angina) from 158 tertiary hospitals and 82 secondary hospitals in 30 provinces, municipalities, and autonomous regions in China. By December 2019, 65,618 patients with STEMI were enrolled. Each hospital was required to consecutively enroll the first 10–30 patients with ACS admitted to the hospital in each month. More details are provided in the published methodological article (10). The study was approved by the Ethics Committee of Beijing Anzhen Hospital, Capital Medical University (approval no. 2014018) with a waiver for informed consent. The study was registered at ClinicalTrials.gov (registration no. NCT02306616).

**FIGURE 1:**
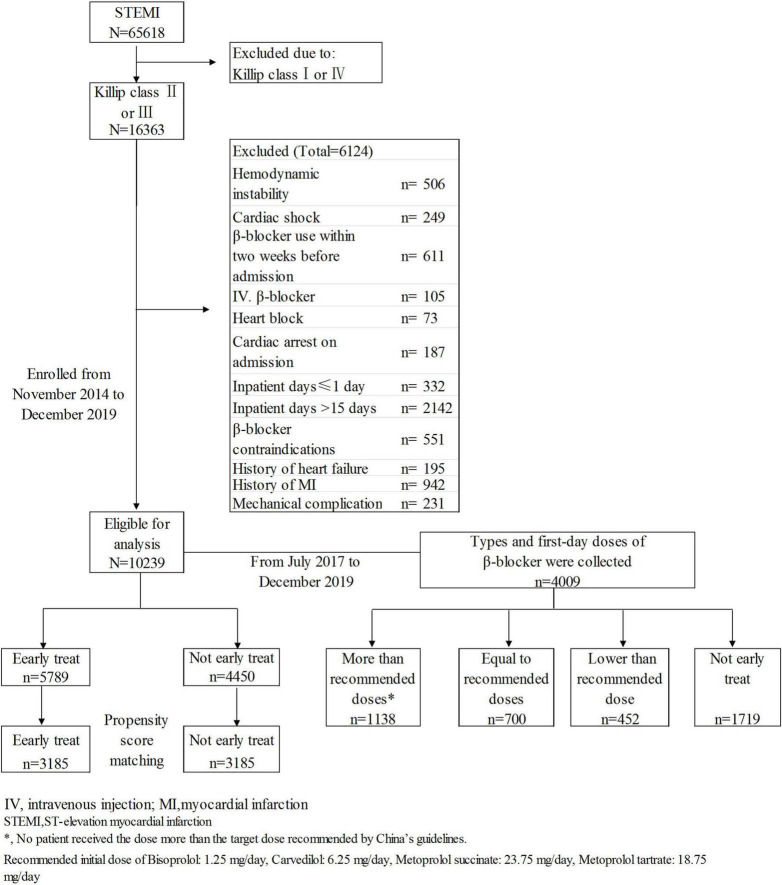
Flowchart of the enrollment process.

Patients with STEMI with mild-moderate HF at admission were selected from the CCC project database. Inclusion criteria: discharge diagnosis of STEMI and Killip class II or III HF at admission. Exclusion criteria: (1) cardiogenic shock or cardiac arrest at admission; (2) hemodynamic instability [systolic blood pressure (SBP) < 85 mmHg or heart rate < 50 beats/minute] at admission; (3) contraindications to β-blocker therapy including second- or third-degree atrioventricular block, bradycardia and asthma; 4) history of HF, myocardial infarction, or revascularization; 5) use of a β-blocker within 2 weeks before admission or in-hospital intravenous injection of a β-blocker; 6) mechanical complications (ventricular septal perforation, papillary muscle rupture, myocardial rupture); (7) length of stay ≤1 day or >15 days; (8) unknown main discharge diagnosis and/or survival status at discharge. A total of 105 patients who received early intravenous β-blocker administration were excluded to prevent the potential impact of this intravenous administration on the effects of early oral β-blocker therapy. The participants with history of myocardial infarction were excluded to ensure the reduced cardiac function at admission was mainly caused by the current myocardial infarction attack. A final total of 10,239 subjects were enrolled (Figure 1). STEMI was defined in accordance with the guidelines issued by the Chinese Society of Cardiology for the diagnosis and management of patients with STEMI (11). The diagnosis was based on myocardial enzyme levels (troponin I or troponin T), ischemia-related clinical symptoms, and electrocardiogram results.

### Data Acquisition and Quality Control

The CCC project made a standardized information acquisition questionnaire, developed a clinical trial electronic data acquisition system based on a web browser for remote data acquisition, and saved the data in the Oracle database. The information recorded in the database was extracted from the medical records of patients with or without early β-blocker therapy. Fixed well-trained data abstractors at participating hospitals were responsible for inputting the data online. The information of the study subjects enrolled in the current month had to be inputted online before the middle of the next month, including demographic characteristics, medications for cardiovascular diseases, disease history, symptoms at admission, in-hospital therapy, electrocardiogram findings, blood biochemical test results, discharge diagnosis, and discharge medication. The research team adopted various measures to ensure the accuracy and integrity of data acquisition, such as automatic verification of data outliers, on-site quality inspections, data integrity monitoring, and random sampling checks (10).

### Study Variables

Early oral β-blocker therapy was defined as the initiation of therapy within 24 h after admission. Non-early oral β-blocker therapies included oral β-blocker therapy initiated more than 24 h after admission or no β-blocker therapy administered during hospitalization. When exploring the correlation between early oral β-blocker therapy and in-hospital outcomes, the adjusted factors included sex, age, smoking status, disease history (hypertension, diabetes, stroke, chronic obstructive pulmonary disease, renal insufficiency, and anemia), medication use within 2 weeks before admission (aspirin, statins, angiotensin-converting enzyme inhibitors, and angiotensin receptor blocker), heart rate at admission, SBP at admission, Killip class at admission, creatinine, and hemoglobin at admission, percutaneous coronary intervention, and medication use within 24 h of admission (including β-blockers, aspirin, statins, angiotensin-converting enzyme inhibitors/angiotensin receptor blockers, and clopidogrel/ticagrelor). In accordance with the uniform requirements of the CCC project, data regarding the β-blocker type and first-day dosage were available for participants enrolled after July 2017; this information was used to explore the dose-response relationship between early oral β-blocker therapy and in-hospital outcomes.

Renal insufficiency was defined as a glomerular filtration rate of <60 mL min^–1^⋅1.73 m^–2^. The glomerular filtration rate was calculated using a formula reported in a study of Chinese patients (12). Patients were defined as having hypertension or elevated blood pressure if they in following situations: (1) SBP ≥ 140 mmHg or diastolic blood pressure ≥ 90 mmHg at admission, (2) history of hypertension, (3) antihypertensive drug administration. Patients were defined as having diabetes if they met any of the following criteria: (1) fasting blood glucose ≥ 7.0 mmol/L (126 mg/dL), (2) HbA1c ≥ 6.5%, (3) history of diabetes, (4) being treated for hypoglycemia. Data on medication use within 24 h of admission and discharge medications were collected from the medical records.

### In-Hospital Outcomes

The primary endpoint of the present study was a combined endpoint composed of in-hospital all-cause death, successful cardiopulmonary resuscitation after cardiac arrest, and cardiogenic shock occurring between the second day of hospitalization and discharge. The secondary endpoint only included all-cause death. These endpoint events were verified and extracted from the medical records and discharge diagnoses.

### Statistical Analysis

Continuous variables were presented as mean ± standard deviation. Categorical variables were presented as percentages. The *t*-test, chi-squared test, or Mantel-Haenszel chi-squared test were used to compare the differences in patient characteristics between the early treatment and non-early treatment groups. The Kaplan-Meier method was used to calculate the cumulative incidence of events during hospitalization, and log-rank tests were used to evaluate whether there were significant differences between the two groups.

Stabilized inverse-probability-of-treatment weighting (IPTW), propensity score matching (PSM), and Cox regression analysis were used to adjust the confounding effects and the latter two methods were used for doubly-robust estimation. The propensity score which was the probability of receiving oral β-blocker therapy for each patient was generated from a multivariable logistic regression model with the variables of age, sex, disease history, medication use within 2 weeks before admission, heart rate, SBP, Killip class, myocardium enzyme levels, percutaneous coronary intervention, and medical treatment received within 24 h of admission. For the IPTW analysis, the contribution of each participant in the analysis was weighted by the stabilized weights. The stabilized weight of each individual in the early treatment group was calculated as the overall rate of early β-blocker therapy/(1/individual propensity score), while the stabilized weight for individual in the non-early treatment group was calculated as (1 – overall rate of early β-blocker therapy)/[1/(1 – individual propensity score)] (13). IPTW with trimming extreme weights at the 1st and 99th percentiles and trimming at the 5th and 95th percentiles were done to evaluate whether the large weights for individual patients had substantial effect on the relationship between the early treatment and in-hospital outcomes (14).

For PSM, patients with and without early β-blocker treatment were matched in a 1:1 ratio based on propensity scores using the nearest neighbor matching algorithm with a caliper of 0.02 and without replacement. In other words, for each early treated patient, untreated patients whose propensity scores were ±0.02 of the score of the early treated patient were considered in the matching process. The untreated patient whose score was closest to the score of the early treated patient was selected as a match. Successfully matched patients were not included in the subsequent matching process.

The myocardial enzyme level was classified as <5, 5–10, and >10 times the local laboratory upper limit of normal for the troponin I or troponin T values. In the dose-response relationship analysis (*n* = 4,009), data regarding the β-blocker dosage received on the first day were used to divide the patients into four groups: those who did not receive early β-blocker therapy (*n* = 1,719), and those who received a β-blocker dosage lower than (*n* = 452), equal to (*n* = 700), or more than (*n* = 1,138) the dosage recommended by the Chinese guidelines for the diagnosis and treatment of heart failure (Supplementary Table 1). A two-sided *P* value of <0.05 was considered significant. All statistical analyses were performed using SAS version 9.4 software (SAS Institute, Cary, NC, United States).

## Results

### Rate of Early Oral β-Blocker Therapy

A total of 10,239 patients with STEMI with Killip class II or III HF at admission were enrolled, of whom 56.5% were in the early treatment group. The rate of early oral β-blocker therapy did not significantly differ between males and females (57.1 vs. 54.9%, χ^2^ = 3.499, *P* = 0.061). More data of the early therapy rate of patients with different characteristics were shown in Supplementary Table 2.

### Characteristics of Patients With and Without Early β-Blocker Therapy

Compared with the non-early treatment group, the early treatment group were younger (average age 63.1 ± 12.8 vs. 65.3 ± 12.4 years, *t* = 8.990, *P* < 0.001, Table 1), had a higher prevalence of hypertension (50.4 vs. 47.0%, χ^2^ = 11.996, *P* < 0.001), and a lower prevalence of renal dysfunction (0.9 vs. 1.4%, *P* = 0.014, Table 1). In addition, the early treatment group had a higher average SBP (130.7 ± 22.2 vs. 128.4 ± 23.4 mmHg) and heart rate (82.5 ± 15.5 vs. 78.8 ± 16.9 beats/minute) at admission and a lower proportion of patients with Killip class III HF (15.7 vs. 19.6%, χ^2^ = 27.084, *P* < 0.001) than the non-early treatment group.

**TABLE 1:** Characteristics of patients with and without early oral β-blocker therapy.

	Before IPTW				After IPTW	
	Early treatment *n* = 5789	Not early treatment *n* = 4450	Statistic value	*P*-value	Early treatment *n* = 5789	Not early treatment *n* = 4450
Female (%)	23.6	25.2	3.499	0.061	24.8	24.3
Age (year)	63.1 ± 12.8	65.3 ± 12.4	8.990	<0.001	64 ± 12.8	64.1 ± 12.8
Age ≥ 70 years (%)	32.5	39.4	50.865	<0.001	35.6	36.3
Smoker (%)	43.7	40.2	12.278	<0.001	50.0	49.2
**Disease history**						
Stroke (%)	7.3	7.8	0.901	0.342	7.7	7.7
Hypertension or elevated blood pressure (%)	50.4	47.0	11.996	<0.001	50.0	49.1
Diabetes (%)	19.5	19.9	0.304	0.581	19.7	19.9
COPD (%)	1.2	1.8	6.414	0.011	1.4	1.5
Renal insufficiency (%)	0.9	1.4	6.085	0.014	1.1	1.1
Anemia (%)	1.4	2.0	5.626	0.018	1.8	1.8
**Preadmission medication**						
Aspirin (%)	12.8	12.7	0.019	0.891	12.5	12.9
Statins (%)	6.1	8.6	22.644	<0.001	7.2	7.3
ACEI (%)	2.3	2.7	1.576	0.209	2.4	2.5
ARB (%)	2.6	2.1	2.728	0.099	2.3	2.4
**Status on admission**						
Heart rate (beats/min)	82.5 ± 15.5	78.8 ± 16.9	11.360	<0.001	81.5 ± 16.1	81.5 ± 18.1
**Heart rate categories (%)**						
50–79 beats/min	46.9	56.2			50.4	50.1
80–109 beats/min	47.4	38.1	92.422	<0.001	44.0	41.7
≥110 beats/min	5.7	5.7			5.6	8.3
SBP (mmHg)	130.7 ± 22.2	128.4 ± 23.4	5.210	<0.001	130.0 ± 22.6	129.7 ± 23.2
**SBP categories (%)**						
85–119 mmHg	31.7	37.5			34.0	34.6
120–139 mmHg	35.7	33.4	37.915	<0.001	34.0	34.1
≥140 mmHg	32.6	29.1			32.0	31.3
Hemoglobin (g/L)	130.7 ± 22	127.9 ± 21.9	6.220	<0.001	129.6 ± 22.3	128.7 ± 22.1
eGFR (ml/min)	100.3 (75.1,125.4)	103.1 (78.6,128.4)	4.127	<0.001	103.2 ± 42.2	103.1 ± 43.9
**eGFR categories (%)**						
≥90 ml/min	65.0	60.7			63.0	61.8
89–60 ml/min	22.3	23.6	24.921	<0.001	23.5	23.4
<60 ml/min	12.7	15.6			13.5	14.8
Killip III (%)	15.7	19.6	27.084	<0.001	18.1	17.1
**Elevated cardiac enzyme (×local laboratory ULN)**						
<5 (%)	17.5	16.8	3.327	0.068	17.8	17.6
5–10 (%)	6.0	4.6			5.2	5.3
≥10 (%)	76.5	78.6			77.0	77.1
**Infarct site**						
Anterior MI (%)	51.9	39.0	168.330	<0.001	46.2	46.4
Inferior MI (%)	27.6	38.6	140.561	<0.001	31.9	32.0
Anteroseptal MI (%)	24.4	18.9	44.597	<0.001	21.8	22.4
Lateral MI (%)	24.5	24.4	0.017	0.895	24.5	24.6
**In-hospital treatment**						
PCI treatment (%)	75.3	73.5	4.070	0.044	74.1	74.1
Aspirin (%)	97.8	92.4	165.799	<0.001	95.5	95.9
Statins (%)	97.4	89.5	274.946	<0.001	93.6	94.0
ACEI (%)	26.8	13.6	262.391	<0.001	20.9	21.4
ARB (%)	37.4	17.1	504.633	<0.001	28.5	28.4
Clopidogrel/ticagrelor (%)	98.4	92.4	223.230	<0.001	95.6	95.9

*ACEI, angiotensin-converting enzyme inhibitor; ARB, angiotensin receptor blocker; COPD, chronic obstructive pulmonary disease; eGFR, estimated glomerular filtration rate; IPTW, inverse-probability-of-treatment weighting; MI, myocardial infarction; PCI, percutaneous coronary intervention; SBP, systolic blood pressure; ULN, upper limit of normal.*

### Association Between Early Oral β-Blocker Therapy and In-Hospital Outcomes

**FIGURE 2:**
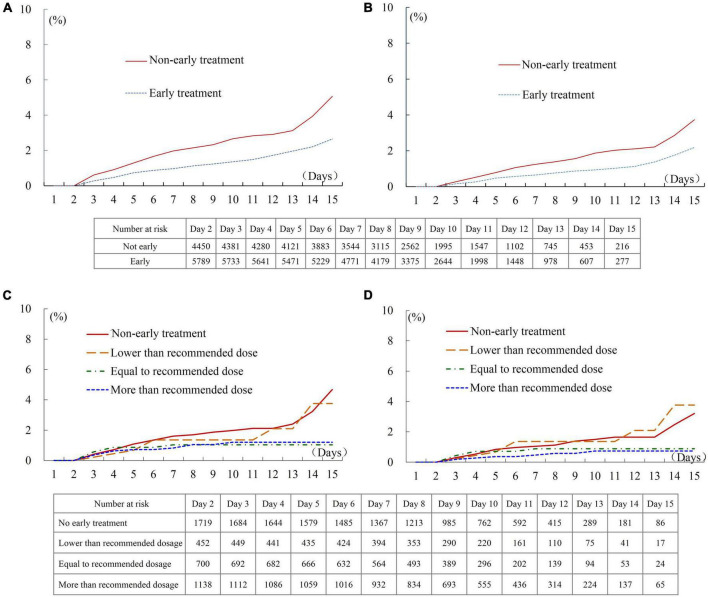
In-hospital outcomes of patients treated with different first-day oral β-blocker dosage. **(A)** Combined endpoint of patients with and without early therapy. **(B)** All-cause death of patients with and without early therapy. **(C)** Combined endpoint of patients treated with different dosages. **(D)** All-cause death of patients treated with different dosages. Recommended initial dosage of Bisoprolol: 1.25 mg/day, Carvedilol: 6.25 mg/day, Metoprolol succinate: 23.75 mg/day, and Metoprolol tartrate: 18.75 mg/day.

The Kaplan-Meier survival analysis showed that the incidence of the combined endpoint event (2.7 vs. 5.1%, *P* < 0.001, Figure 2A) and all-cause mortality (2.2 vs. 3.7%, *P* < 0.001, Figure 2B) were significantly lower in the early treatment group than the non-early treatment group. The association between early oral β-blocker therapy and the risk of in-hospital outcomes was analyzed using IPTW weighted Cox regression analysis. After IPTW, the absolute standard deviations of the characteristics and therapy status of the participants in the two groups were all less than 10% (Supplementary Figure 1). The characteristics of the two groups after IPTW are shown in Table 1. The post-IPTW results indicated that the early treatment group had significantly lower risks of the combined endpoint event (HR = 0.641, 95% CI: 0.486–0.844, *P* = 0.002) and all-cause mortality (HR = 0.687, 95% CI: 0.493–0.958, *P* = 0.027) than the non-early treatment group (Table 2).

**TABLE 2:** Multivariate analysis of the effects of early oral β-blocker therapy.

	After IPTW			Cox regression*			Propensity score matching		
	HR	95%CI	*P* value	HR	95%CI	*P* value	HR	95%CI	*P* value
Combined endpoint^†^	0.641	(0.486–0.844)	0.002	0.665	(0.496–0.894)	0.007	0.633	(0.453–0.884)	0.007
Death	0.687	(0.493–0.958)	0.027	0.584	(0.416–0.821)	0.002	0.650	(0.432–0.979)	0.039

*Sex, age, smoke status, disease history (stroke, hypertension, diabetes, chronic obstructive pulmonary disease, renal insufficiency, and anemia), medicines use (β-blocker, aspirin, statins, and angiotensin-converting enzyme inhibitor/angiotensin receptor blocker) within 2 weeks before admission, heart rate at admission, SBP at admission, Killip class at admission, highest level of myocardium enzyme during admission, infarct site, percutaneous coronary intervention, medical treatment (aspirin, statins, angiotensin-converting enzyme inhibitor/angiotensin receptor blocker, and clopidogrel/ticagrelor) received within 24 h of on-admission were adjusted in the cox regression analysis.

*^†^In-hospital all-cause mortality, successful cardiopulmonary resuscitation after cardiac arrest, and cardiogenic shock.*

*CI, confidence interval; HR, hazard ratio; IPTW, inverse-probability-of-treatment weighting.*

The results of IPTW weighted Cox regression with trimming the extreme weights at the 1st and 99th percentiles (combined endpoint event: HR = 0.691, 95% CI: 0.531–0.898, *P* = 0.006) and trimming at the 5th and 95th percentiles (combined endpoint event: HR = 0.687, 95% CI: 0.521–0.908, *P* = 0.008) show no substantial effect of the large weights for individual patients on the relationship between the early treatment and in-hospital outcome (Table 3).

**TABLE 3:** Results of the inverse-probability-of-treatment weighting analysis with trimming.

Methods	Combined endpoint*			Death		
	HR	95%CI	*P* value	HR	95%CI	*P* value
Trimming at the 1st and 99th percentiles	0.691	(0.531–0.898)	0.006	0.637	(0.454–0.893)	0.009
Trimming at the 5th and 95th percentiles	0.687	(0.521–0.908)	0.008	0.633	(0.443–0.903)	0.012

*In-hospital all-cause mortality, successful cardiopulmonary resuscitation after cardiac arrest, and cardiogenic shock.

*CI, confidence interval; HR, hazard ratio.*

In PSM analysis, 3,185 pairs of participants were matched, the early treatment group also showed lower risks of the in-hospital combined endpoint event (HR = 0.633, 95% CI: 0.453–0.884, *P* = 0.007) and all-cause death (HR = 0.650, 95% CI: 0.432–0.979, *P* = 0.039, Table 2). The histogram of propensity scores was shown in Supplementary Figure 2). Furthermore, the result of multivariate Cox regression analysis, after adjustment for the factors in Table 1, reconfirmed the association between early treatment and lower risks of the in-hospital combined endpoint event (HR = 0.665, 95% CI: 0.496–0.894, *P* = 0.007) and all-cause death (HR = 0.584, 95% CI: 0.416–0.821, *P* = 0.002, Table 2 and Supplementary Tables 3, 4).

In the dose-response relationship analysis of the 4,009 participants enrolled after July 2017, 2,290 patients (57.1%) received early oral β-blocker therapy, while 1,719 (42.9%) patients did not receive early therapy. A dose-response trend between the first-day β-blocker dosage and adverse outcomes was observed. The incidences of the combined endpoint and all-cause death in patients treated with a dosage lower than the recommended dosage were similar to those in patients without early therapy (Figures 2C,D). The incidence of in-hospital outcomes tended to be lower in patients treated with a dosage equal to or greater than the recommended dosage than in patients without early therapy, although these intergroup differences did not reach statistical significance (Log-rank *P* = 0.082 for the combined endpoint, and *P* = 0.079 for all-cause death). The result of multivariate Cox regression analysis showed that the HR of the in-hospital combined endpoint was 1.096 (95% CI: 0.505–2.382, *P* = 0.816), 0.674 (95% CI: 0.296–1.534, *P* = 0.347), and 0.578 (95% CI: 0.296–1.126, *P* = 0.107) in patients who received lower than, equal to, and more than the recommended dosage, respectively, compared with the patients without early therapy (Supplementary Table 5). The results of the analysis performed after combining the groups of patients treated with β-blocker dosages equal to and more than the recommended dosage are shown in Supplementary Figure 3 and Supplementary Table 6.

**FIGURE 3:**
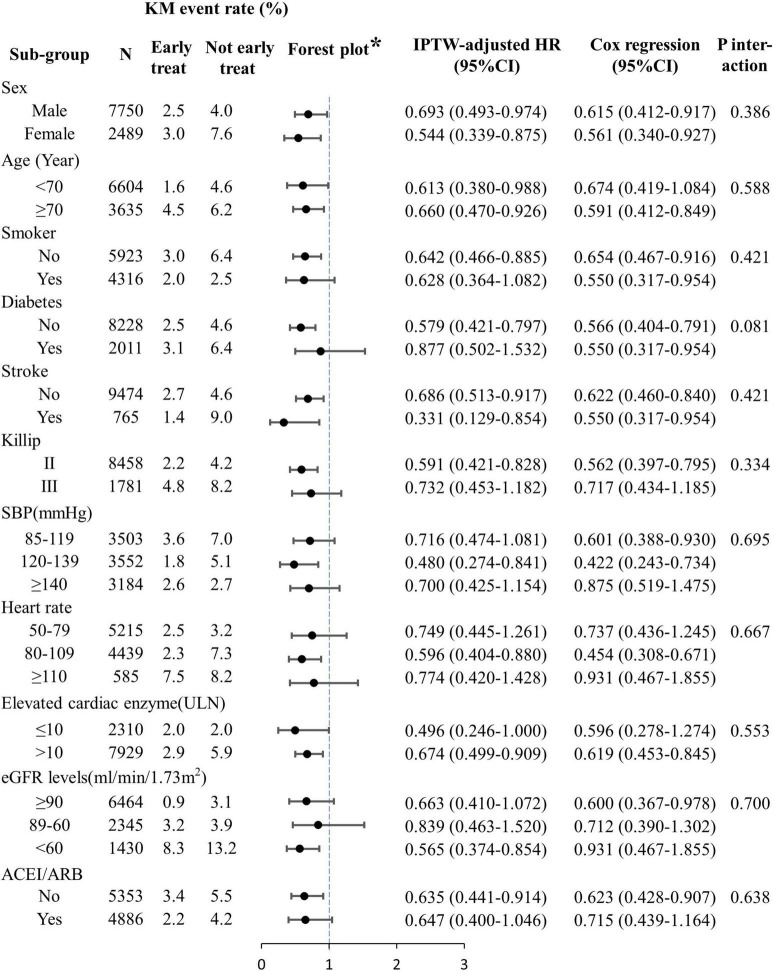
Subgroup analyses of the effects of early oral β-blocker therapy on in-hospital outcomes. ACEI, angiotensin-converting enzyme inhibitor; ARB, angiotensin receptor blocker; eGFR, estimated glomerular filtration rate; IPTW, inverse-probability-of-treatment weighting; PCI, percutaneous coronary intervention; SBP, systolic blood pressure; ULN, upper limit of normal. *IPTW analysis result.

Subgroup analyses indicated that the association between early oral β-blocker therapy and the combined endpoint event was independent of sex, age, disease history, Killip class at admission, pre-hospital medication, and medication used within 24 h of admission (Figure 3). The same results were obtained by PSM (Supplementary Table 7) and multivariate Cox regression analysis (Figure 3).

## Discussion

In the present study cohort, early oral β-blocker therapy was administered to 56.5% of Chinese patients with STEMI with Killip class II or III HF at admission. IPTW, multivariate Cox regression, and PSM analyses consistently found that early oral β-blocker therapy was independently associated with a lower incidence of the combined endpoint event (all-cause death, successful resuscitation after sudden cardiac arrest, or cardiogenic shock) in patients with STEMI with Killip class II or III HF at admission.

Few studies have evaluated the effect of early β-blocker therapy on the outcomes of patients with AMI with Killip class II or III HF at admission. The *post hoc* analysis of the COMMIT randomized controlled trial showed that compared with the placebo group, patients with AMI who received β-blocker therapy within 24 h of admission (5–15 mg intravenously followed by 200 mg/day orally on day 1 until hospital discharge, death, or for 4 weeks) had similar risks of all-cause death (OR = 0.99, 95% CI: 0.92–1.05) and the combined endpoint event in the acute phase (OR = 0.96, 95% CI: 0.90–1.01), but had an increased risk of cardiogenic shock (OR = 1.30, 95% CI: 1.19–1.41) (7). Furthermore, as the Killip class increased, the early β-blocker therapy was in connection with increased risk of all-cause mortality in the acute phase (Killip I: OR = 0.95, Killip III: OR = 1.19) and cardiogenic shock (Killip I: OR = 1.25, Killip III: OR = 1.58) (7). After excluding patients with a length of stay of ≤1 day, the study found that early β-blocker therapy reduced the incidence of all-cause death (4.3 vs. 4.5%) and cardiogenic shock (1.3 vs. 1.6%) (7). However, the study did not analyze the effect of early β-blocker therapy in patients with Killip class II or III HF after excluding those with a length of stay of ≤1 day. Another registry study that performed a multivariate analysis of 1,012 patients with NSTEMI with Killip class II or III HF showed that patients who received early oral β-blocker therapy within 24 h of admission (sometimes accompanied by intravenous therapy) had reduced in-hospital mortality compared with those who did not receive therapy within 24 h of admission (OR = 0.39, 95% CI: 0.23–0.66) (8). The present study performed IPTW, PSM, and multivariate Cox regression analyses of data from patients with STEMI with Killip class II or III HF. The results consistently indicated that patients with early β-blocker therapy had a lower all-cause mortality risk and lower incidence of the combined endpoint event compared with those without early therapy.

Current studies of β-blocker therapy in patients with AMI and Killip class II or III HF differ in design and treatment regimen (7, 8). The COMMIT study was a randomized controlled trial that compared the effect of intravenous and oral β-blocker administration (5–15 mg intravenously initially, followed by 200 mg/day orally on day 1 until hospital discharge, death, or for 4 weeks) with placebo control (7). The GRACE study was a registry study that analyzed the association of early (within 24 h of admission) oral or intravenous β-blocker therapy compared with non-early therapy (8). A *post hoc* analysis of a large-sample (*n* = 87,995) clinical trial showed increased in-hospital risks of all-cause death (RR = 1.70) and cardiogenic shock (RR = 1.48) in patients with intravenous followed by oral β-blocker administration compared with those with early oral β-blocker therapy alone (15). Therefore, it remains unclear whether oral β-blocker therapy alone improves the in-hospital outcomes of patients with AMI with mild-to-moderate HF. In the present study, after excluding patients who received intravenous β-blocker therapy, the risk of adverse events (death, cardiac arrest, or cardiogenic shock) was lower in patients with STEMI and Killip class II or III HF who received early oral β-blocker therapy than in those who did not receive early oral β-blocker therapy.

Current recommendations regarding the initiation of β-blocker therapy for patients with HF with or without myocardial infarction in China, the United States, and Europe are classified separately for the acute and non-acute phases (3, 4, 9, 16–18). The recommendations for therapy in the non-acute phase are consistent, suggesting that patients without contraindications or intolerances to β-blockers should be treated with β-blocker therapy (16–19). However, the recommended timing of the initiation of β-blocker therapy varies between guidelines. Some guidelines recommend starting β-blocker therapy for patients with HF with reduced ejection fraction when the disease condition is relatively stable (18, 19). These recommendations are based on evidence of the effect of such treatment on long-term outcomes in patients with chronic HF [chronic HF (20, 21), outpatient HF (22), and HF at least 2 months post-diagnosis (23–25)]. Guidelines in China and the United States recommend the initiation of β-blocker therapy as early as possible after hemodynamic stabilization (e.g., SBP ≥ 85 mmHg and heart rate ≥ 50 beats/minute) (3, 4) while ESC guideline do not specify an initiation timepoint (16). Therefore, clarifying the effect of β-blocker therapy on patients with AMI and HF in the acute phase will help to unify the recommendations regarding the timing of the initiation of β-blocker therapy. A recent published registry study of hospitalized patients with acute decompensated HF (excluding those with ACS) showed that compared with patients without in-hospital β-blocker therapy, the risk of in-hospital death was 57% lower in those who received low-dose (carvedilol equivalent dose < 10 mg/day) β-blocker therapy (HR: 0.47, 95% CI: 0.27–0.68) and 65% lower in those who received high-dose (carvedilol equivalent dose ≥ 10 mg/day) β-blocker therapy (HR: 0.35, 95% CI: 0.19–0.61) (26). The present study also indicated a similar percent change in HR when comparing the group treated with a first-day β-blocker dosage of more than the recommended dosage with the group treated with a first-day β-blocker dosage of equal to the recommended dosage in patients with STEMI and Killip class II or III HF (the majority of patients with HF).

Our results show that doctors seemed to be less inclined to initiate early β-blocker therapy in patients with inferior myocardial infarction than in those with anterior myocardial infarction (Table 1 and Supplementary Table 2). Inferior STEMI is often due to acute occlusion of the right coronary artery, and the sinus node artery originates from the proximal segment of the right coronary artery. Therefore, the sinus node function may be impaired in patients with inferior STEMI, resulting in a low heart rate, which may affect the treatment decision (27). Our results show that patients with anterior myocardial infarction could benefit from oral β-blocker therapy (HR: 0.642, 95% CI: 0.442–0.933, *P* = 0.020, Supplementary Figure 4). The decreased incidence of the combined endpoint in patients with inferior myocardial infarction did not reach statistical significance (HR: 0.824, 95% CI: 0.463–1.466, *P* = 0.510). Further study is warranted to investigate the use of early β-blocker therapy in patients with inferior STEMI.

Patients with myocardial infarction who had a prolonged length of stay are likely to have more comorbidities, which might result in a high risk of unmeasured or unknown confounders (28, 29). Therefore, the patients with a length of stay of >15 days were excluded. However, an analysis that included the 2,142 excluded patients showed a consistent result.

## Limitations

The CCC project collected data of patients with or without oral β-blocker therapy within 24 h of admission, but it did not record the duration and daily dosage of β-blocker therapy during hospitalization. If the data of the daily β-blocker dosage during hospitalization were available, we could further evaluate the treatment patterns of early β-blocker on in-hospital outcomes. *N*-terminal pro–B-type natriuretic peptide (NT-proBNP) is an important factor affecting the outcomes of patients with HF. The NT-proBNP level collected in CCC project, which were mainly measured during hospitalization and after the clinical decision to administer β-blocker therapy, were available for 79.4% of the subjects included in the present study. Although NT-proBNP was not included in the main analysis, an additional analysis was done of patients with NT-proBNP data; multivariate analysis indicated that the association of early oral β-blocker therapy with a low risk of poor outcomes still existed after adjusting for NT-proBNP level (Supplementary Tables 8, 9).

The present study did not have the left ventricular ejection fraction measured at admission, and therefore the patients cannot be grouped for analysis in accordance with their left ventricular ejection fraction value. The early treatment group were younger, had a higher prevalence of hypertension, a lower prevalence of renal dysfunction, a higher average SBP and heart rate at admission, and a lower proportion of patients with Killip class III HF than the non-early treatment group. This might be because the doctors thought that patients with these characteristics could benefit from or be better able to tolerate the treatment; this issue warrants further investigation. Residual measured and unmeasured confounding may exist owing to the observational nature of the study. These findings may not generalize to patients and hospitals that differ from those in the CCC project.

## Conclusion

Evidence from observational study show patients with STEMI with Killip class II or III HF who receive early oral β-blocker therapy show a lower risk of a combined endpoint composed of death, resuscitation after cardiac arrest, or cardiac shock during hospitalization.

## Data Availability Statement

The data, analytic methods, and study materials will be made available for on-site audit by third parties for purposes of reproducing the results or replicating the procedure.

## Ethics Statement

The studies involving human participants were reviewed and approved by Ethics Committee of Beijing Anzhen Hospital, Capital Medical University. Written informed consent for participation was not required for this study in accordance with the national legislation and the institutional requirements.

## Author Contributions

DZ, SYZ, and MW designed the study. JL, YCH, and NY engaged in data collection and verified the accuracy and completeness of the data. DZ, SYZ, MW, JL, SS, YH, GF, JBG, CSM, YLH, LM, and TL interpreted the data. MW analyzed the data and wrote the first draft of the manuscript. DZ, SYZ, GF, SS, and TL critically reviewed and revised the manuscript and other authors provided very valuable comments for manuscript revision. All authors approved this manuscript and agreed to be accountable for all aspects of work ensuring integrity and accuracy.

## Conflict of Interest

GF reports consulting for Abbott, Amgen, AstraZeneca, Bayer, Cytokinetics, Janssen, Medtronic, Merck, and Novartis. The remaining authors declare that the research was conducted in the absence of any commercial or financial relationships that could be construed as a potential conflict of interest.

## Publisher’s Note

All claims expressed in this article are solely those of the authors and do not necessarily represent those of their affiliated organizations, or those of the publisher, the editors and the reviewers. Any product that may be evaluated in this article, or claim that may be made by its manufacturer, is not guaranteed or endorsed by the publisher.
