# Supplementary material for: Association Between Early Oral β-Blocker Therapy and In-Hospital Outcomes in Patients With ST-Elevation Myocardial Infarction With Mild-Moderate Heart Failure: Findings From the CCC-ACS Project
Source: Front Cardiovasc Med. 2022 Apr 15;9:828614. doi: 10.3389/fcvm.2022.828614 (PMC9051227; doi:10.3389/fcvm.2022.828614)
Supplement: Supplementary file 1 [file Data_Sheet_1.pdf]

Supplement Materials

Contents

Supplement Tables **1**

Supplement Table 1 Distribution of the first-day dose of β-blocker in patients received early treatment 1

Supplement Table 2 Early oral β-blocker treatment rate among patients with different characters 2

Supplement Tables 3 Multivariable Cox regression analysis of association between early oral β-blocker treatment and in-hospital combined endpoint 5

Supplement Tables 4 Multivariable Cox regression analysis of association between early oral β-blocker treatment and in-hospital death 6

Supplement table 5 Multivariable Cox regression analysis of dose-response relationship among early oral β-blocker treatment (first-day doses) and in-hospital combined endpoint 7

Supplement table 6 Multivariable Cox regression analysis of dose-response relationship among early oral β-blocker treatment (first-day doses) and in-hospital combined endpoint after combining patients treated with equal to and more than the recommended dosages 8

Supplement Table 7 Sub-group analysis of Cox regression analysis of association between early oral β-blocker treatment and in-hospital combined endpoint after propensity score matching 9

Supplement table 8 Multivariable Cox regression analysis of association between early oral β-blocker treatment and in-hospital combined endpoint after adjusting the NT-proBNP levels 11

Supplement table 9 Multivariable Cox regression analysis of association between early oral β-blocker treatment and in-hospital death after adjusting the NT-proBNP levels 12

Supplement figures 13

Supplemental Figure 1 Absolute standardized differences before and after inverse probability of treatment weighting 13

Supplemental Figure 2 Histogram of propensity scores 14

Supplemental Figure 3 In-hospital outcomes of patients treated with different doses of β-blocker during first day of treatment after combining patients treated with equal to and more than the recommended dose 15

Supplemental Figure 4 Subgroup analyses of the effects of early oral β-blocker therapy on in-hospital outcomes among patients with different infarction site 16

Supplement Tables

Supplement Table 1 Distribution of the first-day dose of β-blocker in patients received early treatment

Type	**Lower than recommended dose (mg/day)**<br>Bisoprolol:<1.25<br>Carvedilol: <6.25<br>Metoprolol succinate: <23.75<br>Metoprolol tartrate: <18.75	**Equal to recommended dose(mg/day)**<br>Bisoprolol:=1.25<br>Carvedilol: =6.25<br>Metoprolol succinate: =23.75<br>Metoprolol tartrate: =18.75	**More than recommended dose(mg/day)***<br>Bisoprolol:>1.25<br>Carvedilol: >6.25<br>Metoprolol succinate: >23.75<br>Metoprolol tartrate: >18.75	**Overall**
Bisoprolol N (%)	8 (2.7%)	44 (14.7%)	248 (82.7%)	300 (100%)
Carvedilol N (%)	1 (33.3%)	1 (33.3%)	1 (33.3%)	3 (100%)
Metoprolol succinate N (%)	105 (9.3%)	646 (57.1%)	381 (33.7%)	1132 (100%)
Metoprolol tartrate N (%)	338 (39.5%)	9 (1.1%)	508 (59.4%)	855 (100%)
Total N (%)	452 (19.7%)	700 (30.6%)	1138 (49.7%)	2290 (100%)

*, No patient received the dose more than the target dose recommended by the Chinese guidelines for the diagnosis and treatment of heart failure 2018.

Supplement Table 2 Early oral β-blocker treatment rate among patients with different characters

	N	Early oral treatment （%）	χ^2 value	P value
**All**	10239	56.5		
**Gender**				
Male	7750	57.1	3.499	0.061
Female	2489	54.9		
**Age group (Year)**				
<50	1490	65.0	79.238	<0.001
50-59	2126	58.5		
60-69	2988	56.6		
70-79	2501	52.7		
≥80	1134	49.8		
**Smoker**				
No	5923	55.1	0.901	0.342
Yes	4316	58.5		
**Disease history**				
**Stroke**				
No	9474	56.7	12.278	<0.001
Yes	765	54.9		
**Hypertension**				
No	5228	54.9	11.996	<0.001
Yes	5011	58.3		
**Diabetes**				
No	8228	56.7	0.304	0.581
Yes	2011	56.0		
**COPD**				
No	10092	56.7	6.414	0.011
Yes	147	46.3		
**Renal insufficiency**				
No	10122	56.7	6.085	0.014
Yes	117	45.3		
**Anemia**				
No	10065	56.7	5.626	0.018
Yes	174	47.7		
**Preadmission medication**				
**Aspirin**				
No	8936	56.5	0.019	0.891
Yes	1303	56.7		
**Statins**				
No	9502	57.2	22.644	<0.001
Yes	737	48.2		

	N	Early oral treatment （%）	χ^2 value	P value
**ACEI**				
No	9981	56.6	1.576	0.209
Yes	258	52.7		
**ARB**				
No	9996	56.4	2.728	0.099
Yes	243	61.7		
**Heart rate (beats/min)**				
51-59	617	30.5	127.680	<0.001
60-79	4598	55.0		
70-109	4439	61.8		
>=110	585	56.6		
**SBP (mmHg)**				
85-119	3503	52.4	23.774	<0.001
120-139	3552	58.1		
140-159	2098	60.4		
>=160	1086	57.2		
**Killip class**				
II	8458	57.7	27.084	<0.001
III	1781	51.0		
**Elevated cardiac enzyme （×local laboratory ULN）**				
< 5	1760	57.5	3.327	0.068
5-10	550	63.1		
≥10	7929	55.9		
**Infarct site**				
Anterior MI				
No	5498	50.6	168.330	<0.001
Yes	4741	63.4		
Inferior MI				
No	6924	60.6	140.561	<0.001
Yes	3315	48.1		
Anteroseptal MI				
No	7987	54.8	44.597	<0.001
Yes	2252	62.7		
Lateral MI				
No	7736	56.5	0.017	0.895
Yes	2503	56.7		
**In-hospital treatment**				
PCI treatment				
No	2609	54.8	4.070	0.044
Yes	7630	57.1		

	N	Early oral treatment （%）	χ^2 value	P value
Aspirin				
No	464	27.6	165.799	<0.001
Yes	9775	57.9		
Statins				
No	622	24.6	12.278	<0.001
Yes	9617	58.6		
ACEI				
No	8085	52.4	262.391	<0.001
Yes	2154	71.9		
ARB				
No	7312	49.6	504.633	<0.001
Yes	2927	73.9		
Clopidogrel/ticagrelor				
No	428	21.5	223.230	<0.001
Yes	9811	58.1		

ACEI, angiotensin converting enzyme inhibitor; ARB, angiotensin receptor blocker; COPD, chronic obstructive pulmonary disease; IPTW, inverse-probability-of-treatment weighting; MI, myocardial infarction; PCI, percutaneous coronary intervention; SBP, systolic blood pressure; ULN, upper limit of normal

Supplement Tables 3 Multivariable Cox regression analysis of association between early oral β-blocker treatment and in-hospital combined endpoint

	P value	HR	95%CI
**Early β-blocker treatment**	0.007	0.665	(0.496-0.894)
**Age (Year)**	<0.001	1.049	(1.034-1.063)
**Heart rate (beats/min)**	<0.001	1.020	(1.013-1.027)
**SBP (mmHg)**			
85-119	--	1.0	--
120-139	0.007	0.625	(0.445-0.878)
140-159	0.006	0.576	(0.391-0.850)
≥160	0.002	0.457	(0.276-0.756)
**Renal insufficiency**	0.037	2.016	(1.042-3.900)
**eGFR levels(ml/min/1.73m^2)**			
≥90	--	1.0	--
89-60	0.407	1.180	(0.798-1.745)
<60	<0.001	2.828	(1.998-4.003)
**Infarct site of MI**			
Inferior	0.005	0.619	(0.443-0.864)
**In-hospital treatment**			
PCI treatment	<0.001	0.401	(0.300-0.535)
ACEI/ARB	0.002	0.611	(0.449-0.832)

ACEI, angiotensin converting enzyme inhibitors; ARB, angiotensin receptor blockers; CI, confidence interval; eGFR, estimated glomerular filtration rate; HR, hazard ratio; PCI, percutaneous coronary intervention; SBP, systolic blood pressure

Combined endpoint include death, successful cardiopulmonary resuscitation after cardiac arrest, or cardiac shock.

The gender, age, smoke status, disease history (stroke, hypertension, diabetes, COPD, renal insufficiency, anemia), medicines use (β-blocker, aspirin, statins, ACEI, ARB or clopidogrel/ticagrelor) within two weeks before admission, heart rate, SBP, Killip class, myocardium enzyme, infarct site, PCI treatment, medical treatment (aspirin, statins, ACEI, ARB or clopidogrel/ticagrelor) received within 24 hours of on-admission were adjusted in the cox regression analysis.

Supplement Tables 4 Multivariable Cox regression analysis of association between early oral β-blocker treatment and in-hospital death

	P value	HR	95%CI
**Early β-blocker treatment**	0.002	0.584	(0.416-0.821)
**Age(Year)**	<0.001	1.054	(1.036-1.071)
**Heart rate(beats/min)**	<0.001	1.021	(1.013-1.029)
**SBP (mmHg)**			
85-119	--	1	--
120-139	0.108	0.725	(0.490-1.073)
140-159	0.019	0.583	(0.372-0.916)
≥160	<0.001	0.223	(0.105-0.474)
**Renal insufficiency**	<0.001	3.447	(1.753, 6.779)
**eGFR levels(ml/min/1.73m^2)**			
≥90	--	1	--
89-60	0.923	1.023	(0.642-1.632)
<60-	<0.001	2.275	(1.503-3.443)
**PCI treatment**	<0.001	0.368	(0.259-0.521)
**Inferior MI**	0.005	0.549	(0.363-0.831)

CI, confidence interval; eGFR, estimated glomerular filtration rate; HR, hazard ratio; PCI, percutaneous coronary intervention; SBP, systolic blood pressure

The gender, age, smoke status, disease history (stroke, hypertension, diabetes, COPD, renal insufficiency, anemia), medicines use (β-blocker, aspirin, statins, ACEI, ARB or clopidogrel/ticagrelor) within two weeks before admission, heart rate, SBP, Killip class, myocardium enzyme, infarct site, PCI treatment, medical treatment (aspirin, statins, ACEI, ARB or clopidogrel/ticagrelor) received within 24 hours of on-admission were adjusted in the cox regression analysis.

Supplement table 5 Multivariable Cox regression analysis of dose-response relationship among early oral β-blocker treatment (first-day doses) and in-hospital combined endpoint

	Combined endpoint				Death			
	Events(n)	P value	HR	95%CI	Events(n)	P value	HR	95%CI
Non-early treatment n=1719	36	--	1	--	27	--	1	--
Lower than recommended dose n=452	8	0.816	1.096	(0.505-2.382)	8	0.356	1.457	(0.655-3.240)
Equal to recommended dose n=700	7	0.347	0.674	(0.296-1.534)	6	0.592	0.782	(0.318-1.920)
More than recommended dose n=1138	12	0.107	0.578	(0.296-1.126)	7	0.096	0.486	(0.208-1.136)

HR, hazard ratio; CI, confidence interval

Sex, age, smoke status, disease history (stroke, hypertension, diabetes, COPD, renal insufficiency, anemia), medicines use (β-blocker, aspirin, statins, ACEI/ARB) within two weeks before admission, heart rate at admission, SBP at admission, Killip class at admission, highest level of myocardium enzyme during admission, infarct site, PCI treatment, medical treatment (aspirin, statins, ACEI/ARB or clopidogrel/ticagrelor) received within 24 hours of on-admission were adjusted in the cox regression analysis.

Supplement table 6 Multivariable Cox regression analysis of dose-response relationship among early oral β-blocker treatment (first-day doses) and in-hospital combined endpoint after combining patients treated with equal to and more than the recommended dosages

	Combined endpoint			Death		
	**P value**	**HR**	**95%CI**	**P value**	**HR**	**95%CI**
No early treatment	--	1	--	--	1	--
Lower than recommended dose	0.818	1.095	(0.504, 2.379)	0.357	1.456	(0.655, 3.238)
Equal or greater than recommended dose	0.086	0.611	(0.348, 1.071)	0.125	0.592	(0.303, 1.156)

HR, hazard ratio; CI, confidence interval

Sex, age, smoke status, disease history (stroke, hypertension, diabetes, COPD, renal insufficiency, anemia), medicines use (β-blocker, aspirin, statins, ACEI/ARB) within two weeks before admission, heart rate at admission, SBP at admission, Killip class at admission, highest level of myocardium enzyme during admission, infarct site, PCI treatment, medical treatment (aspirin, statins, ACEI/ARB or clopidogrel/ticagrelor) received within 24 hours of on-admission were adjusted in the cox regression analysis.

Supplement Table 7 Sub-group analysis of Cox regression analysis of association between early oral β-blocker treatment and in-hospital combined endpoint after propensity score matching

Sub-group	Matched number	HR (96%CI)
**All**	3185 vs 3185	0.633 (0.453-0.885)
**Sex**		
Male	2384 vs 2384	0.706 (0.465-1.071)
Female	767 vs 767	0.604 (0.340-1.074)
**Age（Year）**		
<70	1967 vs 1967	0.627 (0.343-1.145)
≥70	1180 vs 1180	0.574 (0.378-0.873)
**Smoker**		
No	1860 vs 1860	0.630 (0.423-0.938)
Yes	1301 vs 1301	0.420 (0.212-0.830)
**Diabetes**		
No	2540 vs 2540	0.600 (0.410-0.878)
Yes	614 vs 614	0.930 (0.471-1.836)
**Stroke**		
No	2942 vs 2942	0.716 (0.503-1.020)
Yes	220 vs 220	0.229 (0.066-0.799)
**Killip**		
II	2600 vs 2600	0.584 (0.382-0.891)
III	539 vs 539	0.613 (0.344-1.094)
**SBP (mmHg)**		
85-119	1124 vs 1124	0.556 (0.341-0.906)
120-139	1042 vs 1042	0.526 (0.262-1.056)
≥140	967 vs 967	0.730 (0.373-1.428)
**Heart rate (beats/min)**		
50-79	1652 vs 1652	0.721 (0.405-1.285)
80-109	1312 vs 1312	0.535 (0.336-0.850)
≥110	170 vs 170	0.904 (0.384-2.131)
**Elevated cardiac enzyme (ULN)**		
≤10	691 vs 691	0.800 (0.345-1.852)
>10	2439 vs 2439	0.563 (0.390-0.813)
**eGFR levels(ml/min/1.73m^2)**		
≥90	1964 vs 1964	0.615 (0.342-1.104)
89-60	750 vs 750	0.826 (0.424-1.606)
<60	427 vs 427	0.661 (0.383-1.141)

**Anterior MI**		
No	1714 vs 1714	0.480 (0.285-0.810)
Yes	1440 vs 1440	0.747 (0.475-1.176)
**Inferior MI**		
No	2160 vs 2160	0.637 (0.435-0.934)
Yes	1004 vs 1004	0.605 (0.296-1.240)
**Anteroseptal MI**		
No	2482 vs 2482	0.473 (0.316-0.706)
Yes	691 vs 691	0.898 (0.401-2.011)
**Lateral MI**		
No	2399 vs 2399	0.539 (0.363-0.801)
Yes	766 vs 766	0.866 (0.439-1.709)
**ACEI/ARB**		
No	1930 vs 1930	0.564 (0.377-0.845)
Yes	1234 vs 1234	0.774 (0.418-1.433)

ACEI, angiotensin converting enzyme inhibitors; ARB, angiotensin receptor blockers; CI, confidence interval; eGFR, estimated glomerular filtration rate; HR, hazard ratio; PCI, percutaneous coronary intervention; SBP, systolic blood pressure

Supplement table 8 Multivariable Cox regression analysis of association between early oral β-blocker treatment and in-hospital combined endpoint after adjusting the NT-proBNP levels

	P value	HR	95%CI
**Early treatment of β-blocker**	0.006	0.637	(0.461-0.880)
**Quartile of NT-proBNP***			
Q1	0.026	0.472	(0.244-0.913)
Q2	0.712	0.919	(0.586-1.440)
Q3	0.100	0.706	(0.467-1.069)
Q4	--	1	--
**Age (Year)**	<0.001	1.045	(1.029-1.061)
**Renal insufficiency**	0.023	2.171	(1.111-4.243)
**Heart rate (beats/min)**	<0.001	1.021	(1.014-1.029)
**SBP (mmHg)**			
86-119	--	1	--
120-139	0.015	0.631	(0.436-0.913)
140-159	0.006	0.549	(0.359-0.839)
>=160	0.001	0.357	(0.198-0.646)
**eGFR levels(ml/min/1.73m^2)**			
≥90	--	1	--
89-60	0.893	1.030	(0.667-1.592)
<60	<0.001	2.531	(1.711-3.744)
**Inferior MI**	0.003	0.560	(0.385-0.815)
**In-hospital treatment**			
**PCI treatment**	<0.001	0.397	(0.288-0.545)
**ACEI/ARB therapy**	0.002	0.580	(0.412-0.816)

ARB, angiotensin receptor blockers; CI, confidence interval; eGFR, estimated glomerular filtration rate; HR, hazard ratio; PCI, percutaneous coronary intervention; SBP, systolic blood pressure; BNP, B-type natriuretic peptide

*, The quartile of BNP was defined according to the quartile of N-terminal pro-B-type natriuretic peptide (NT-proBNP) and BNP level. For participants both measured the levels of BNP and NT-proBNP, the higher quartile was used to define the variable of quartile of BNP. The three cut-off points of quartile of NT-proBNP were 313.0, 1130.0, and 3241.0 pg/ml, and the three cut-off points of quartile of BNP were 82.5, 293.0, and 927.1 pg/ml.

The gender, age, smoke status, disease history (stroke, hypertension, diabetes, COPD, eGFR, anemia), infarct site, medicines use (β-blocker, aspirin, statins, ACEI, ARB or clopidogrel/ticagrelor) within two weeks before admission, heart rate, SBP, Killip class, myocardium enzyme, PCI treatment, medical treatment (aspirin, statins, ACEI, ARB or clopidogrel/ticagrelor) received within 24 hours of on-admission were adjusted in the cox regression analysis. Except the NT-proBNP and early β-blocker treatment, all other variables were analyzed by stepwise variable selection procedure.

Supplement table 9 Multivariable Cox regression analysis of association between early oral β-blocker treatment and in-hospital death after adjusting the NT-proBNP levels

	P value	HR	95%CI
**Early treatment of β-blocker**	0.001	0.531	(0.364-0.775)
**Quartile of NT-proBNP**			
Q1	0.083	0.503	(0.231-1.094)
Q2	0.250	0.712	(0.400-1.269)
Q3	0.092	0.647	(0.390-1.073)
Q4	--	1	--
**Age (Year)**	<0.001	1.049	(1.030-1.069)
**Renal insufficiency**	<0.001	3.727	(1.868-7.435)
**Heart rate (beats/min)**	<0.001	1.022	(1.013-1.032)
**SBP (mmHg)**			
86-119	--	1	--
120-139	0.088	0.687	(0.447-1.058)
140-159	0.018	0.549	(0.335-0.901)
>=160	<0.001	0.12	(0.043-0.337)
**eGFR levels(ml/min/1.73m^2)**			
≥90	--	1	--
89-60	0.619	0.875	(0.518-1.480)
<60	0.004	1.996	(1.247-3.192)
**Inferior MI**	0.001	0.439	(0.270-0.716)
**PCI treatment**	<0.001	0.382	(0.259-0.564)

ARB, angiotensin receptor blockers; CI, confidence interval; eGFR, estimated glomerular filtration rate; HR, hazard ratio; PCI, percutaneous coronary intervention; SBP, systolic blood pressure; BNP, B-type natriuretic peptide

*,The quartile of BNP was defined according to the quartile of N-terminal pro-B-type natriuretic peptide (NT-proBNP) and BNP level. For participants both measured the levels of BNP and NT-proBNP, the higher quartile was used to define the variable of quartile of BNP. The three cut-off points of quartile of NT-proBNP were 313.0, 1130.0, and 3241.0 pg/ml, and the three cut-off points of quartile of BNP were 82.5, 293.0, and 927.1 pg/ml.

The gender, age, smoke status, disease history (stroke, hypertension, diabetes, COPD, eGFR, anemia), infarct site, medicines use (β-blocker, aspirin, statins, ACEI, ARB or clopidogrel/ticagrelor) within two weeks before admission, heart rate, SBP, Killip class, myocardium enzyme, PCI treatment, medical treatment (aspirin, statins, ACEI, ARB or clopidogrel/ticagrelor) received within 24 hours of on-admission were adjusted in the cox regression analysis. Except the NT-proBNP and early β-blocker treatment, all other variables were analyzed by stepwise variable selection procedure.

Supplement figures

Supplemental Figure 1 Absolute standardized differences before and after inverse-probability-of-treatment weighting

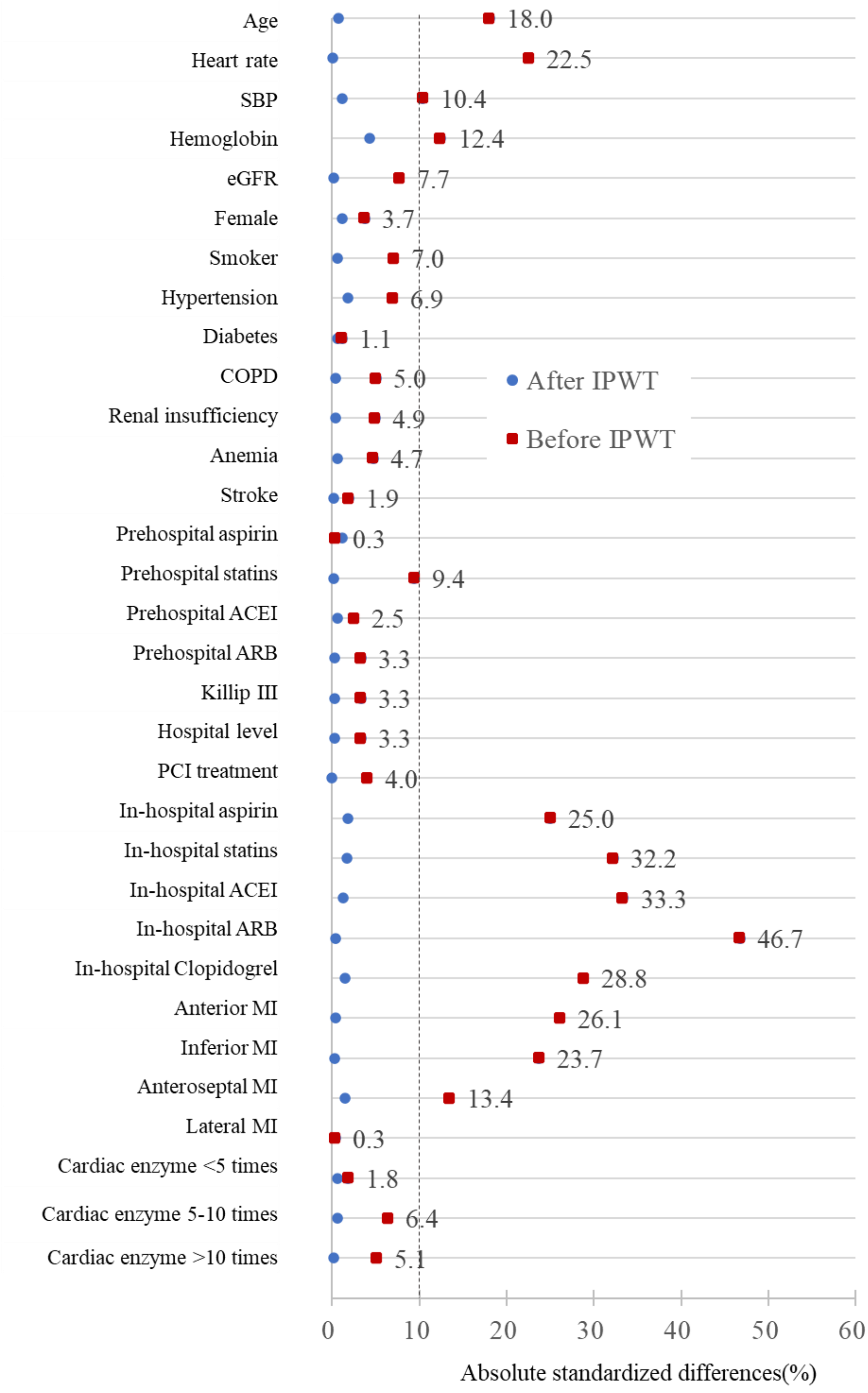

ACEI, angiotensin converting enzyme inhibitor; ARB, angiotensin receptor blocker; COPD, chronic obstructive pulmonary disease; IPTW, inverse-probability-of-treatment weighting; PCI, percutaneous coronary intervention; SBP, systolic blood pressure

Supplemental Figure 2 Histogram of propensity scores

2a Histogram of propensity scores before matching

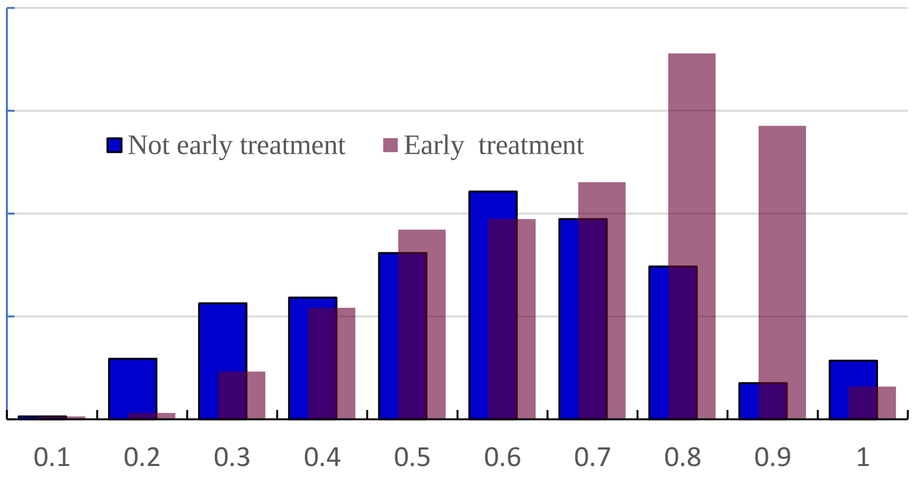

2b Histogram of propensity scores after matching

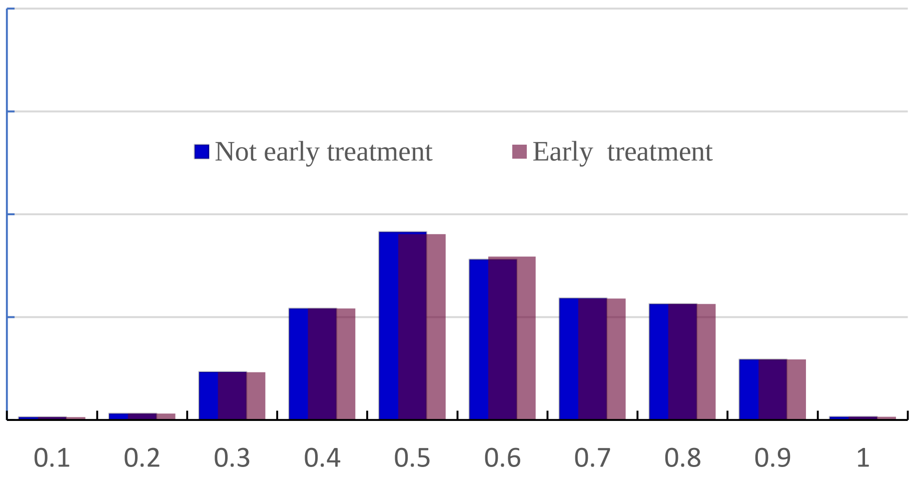

Supplemental Figure 3 In-hospital outcomes of patients treated with different doses of β-blocker during first day of treatment after combining patients treated with equal to and more than the recommended dose

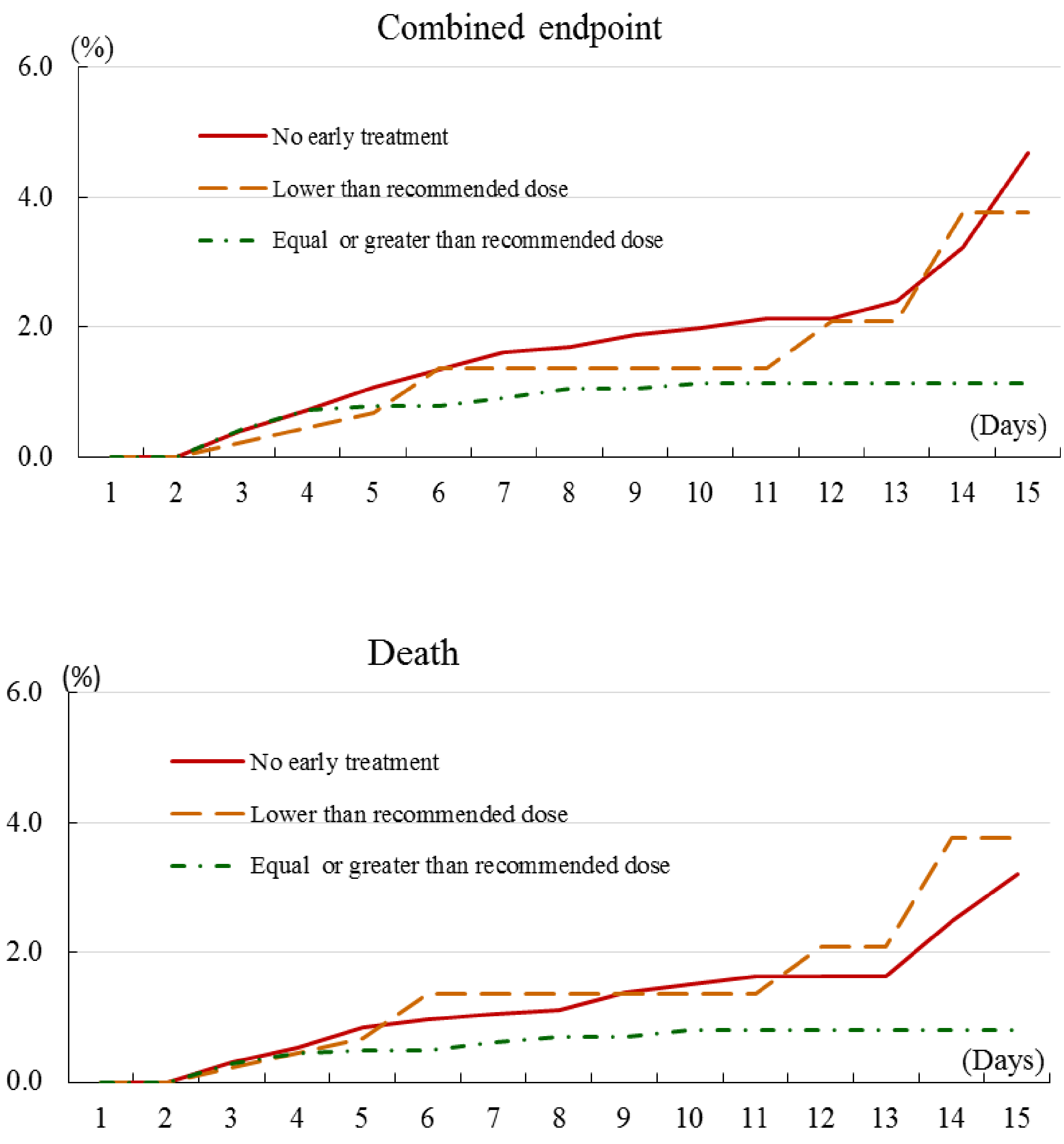

Log rank P for combined endpoint: 0.035, for all-cause death:0.030

Supplemental Figure 4 Subgroup analyses of the effects of early oral β-blocker therapy on in-hospital outcomes among patients with different infarction site

Sub-group	N	KM event rate (%) Early treat	KM event rate (%) Not early treat	Forest plot	IPWT-adjusted HR (95%CI)	P-interaction
**Anterior MI**						
No	5498	2.5	4.7		0.641 (0.422-0.972)	0.761
Yes	4741	2.8	5.6		0.642 (0.442-0.933)	
**Inferior MI**						
No	6924	3.0	6.0		0.592 (0.432-0.812)	
Yes	3315	1.7	3.6		0.824 (0.463-1.466)	0.232
**Anteroseptal MI**						
No	7987	3.0	5.1		0.552 (0.403-0.756)	0.067
Yes	2252	1.8	4.9		1.087 (0.589-2.006)	
**Lateral MI**						
No	7736	2.8	5.1		0.629 (0.457-0.865)	0.266
Yes	2503	2.2	4.9		0.677 (0.392-1.171)	

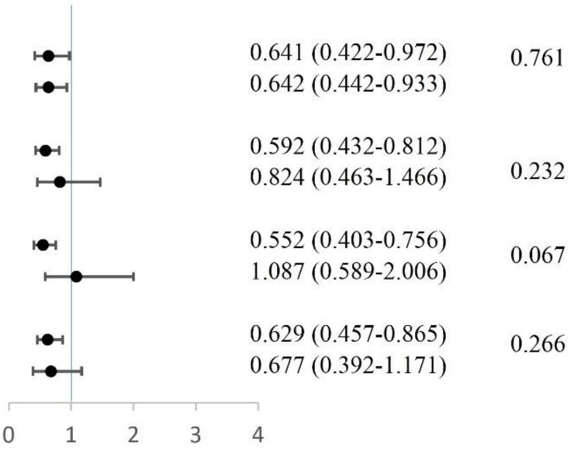

HR, hazard ratio; IPTW, inverse-probability-of-treatment weighting; MI, myocardial infarction
